# Nanohybrids of 2D Black Phosphorus with Phthalocyanines: Role of Interfacial Interactions in Heterostructure Development

**DOI:** 10.1002/chem.202403570

**Published:** 2024-12-10

**Authors:** Doriana Scittarelli, Serena Coiai, Francesca Cicogna, Stefano Legnaioli, Martina Dell'Angela, Alberto Verdini, Roberto Costantini, Manuel Serrano‐Ruiz, Elisa Passaglia, Maria Caporali

**Affiliations:** ^1^ Consiglio Nazionale delle Ricerche Istituto di Chimica dei Composti Organometallici SS Pisa (CNR-ICCOM SS Pisa), Via G. Moruzzi 1 Pisa 56124 Italy; ^2^ Consiglio Nazionale delle Ricerche Istituto Officina dei Materiali (IOM) Area Science Park, Strada Statale 14, km 163.5 Trieste I-34149 Italy; ^3^ Consiglio Nazionale delle Ricerche Istituto Officina dei Materiali (IOM) Via Alessandro Pascoli Perugia I-06123 Italy; ^4^ Dipartimento di Fisica Università di Trieste Via Valerio 2 Trieste I-34127 Italy; ^5^ Consiglio Nazionale delle Ricerche Istituto di Chimica dei Composti Organometallici (ICCOM) Via Madonna del Piano 10 Sesto Fiorentino 50019 Italy

**Keywords:** Black phosphorus (bP), Electron transfer, Manganese, NEXAFS, Phthalocyanine (Pc)

## Abstract

New 2D black phosphorus (bP)–phthalocyanine (Pc) nanohybrids have been synthesized by liquid phase exfoliation of black phosphorus crystals in the presence of two organic dyes: phthalocyanine (Pc) and manganese phthalocyanine (MnPc). The key role of the metal cation in the interfacial interaction between the organic dye and bP nanosheets was demonstrated by X‐ray absorption spectroscopy and associated with an electron transfer between the metal cation Mn^2+^ and bP nanosheets, which resembles a coordinative chemical bond. On the other hand, the interaction between bP nanosheets and pure phthalocyanine is governed by van der Waals forces. The fluorescence of both hybrids is significantly reduced indicating effective separation of the photoinduced charge, implying the formation of a heterojunction between the organic molecules and the bP nanosheets. These findings provide important insights into the interfacial interactions in bP‐Pc nanohybrids that are relevant for application in 2D organic/inorganic devices.

## Graphical abstract

New 2D black phosphorus (bP)–phthalocyanine nanohybrids were prepared by liquid phase exfoliation of bP crystals with phthalocyanine (Pc) and Mn phthalocyanine (MnPc). Spectroscopic analyses revealed the development of heterostructures due to the interaction of bP with the aromatic and heteroaromatic rings of Pc, as well as coordinative bonds between P atoms and the empty d orbitals of Mn^2+^ in MnPc‐bP hybrid.

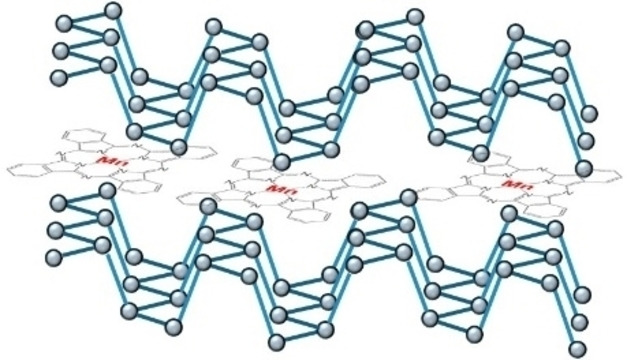

## Introduction

In the frame of sustainable development and the increasing focus on solar‐energy conversion and storage technologies, the study of heterostructures based on organic dyes absorbing light in the Vis–NIR range and integrated with inorganic semiconductors is of great interest to the scientific community.^[1]^ Among organic dyes, metal phthalocyanines^[2]^ have gained great attention due to their conjugated aromatic framework, high thermal stability, and the ability to stabilize various oxidation states of the central metal ion. Hence, several applications have been envisaged as optical, (photo)electronic and spintronic devices, organic photovoltaic cells, in photocatalysis and electrocatalysis for water splitting and carbon dioxide reduction^[2]^ and in the biomedical field as contrast agents for photothermal therapy.^[3]^ In this work, we investigated the synthesis and characterization of novel nanohybrids of phthalocyanine (Pc) and manganese phthalocyanine (MnPc) with few‐layer black phosphorus. The latter is an emerging 2D inorganic semiconductor^[4]^ that has attracted remarkable interest due to its peculiar physicochemical properties as high carrier mobility (up to 1000 cm^2^/V s), high on‐off current ratio (up to 10^4^) and a tunable direct band gap (from 0.3 eV to 2.0 eV). Few nanohybrids of bP with metal phthalocyanines are known in the literature. For example, Wang *et al*.^[5]^ developed a device based on CuPc‐bP to study the interactions at the interface between Pc and bP. The preparation was made by thermal deposition of a layer of Cu(II)Pc on a film of bP previously obtained by mechanical exfoliation. Sarswat *et al*.^[6]^ prepared a sensor for the detection of thiols, through the deposition of CoPc by solution casting from a dimethylformamide (DMF) solution on a film of bP. Lately, other nanohybrids GaPc‐bP,^[7]^ CoPc‐bP^[8]^ and ZrPc‐bP^[9]^ have been studied and have shown to be promising in applications as optical limiting devices^[7]^ and flame retardants respectively.^[9]^

However, no works have reported the properties of the hybrid material formed by the direct intercalation of Pc between bP layers using liquid phase exfoliation (LPE). This work aimed to synthesize Pc‐bP and MnPc‐bP nanohybrids by using LPE and to study their structural and photophysical properties. We show that the presence of a metal cation in the macrocycle can strengthen the interaction with bP.

## Results and Discussions

**Figure 1:**
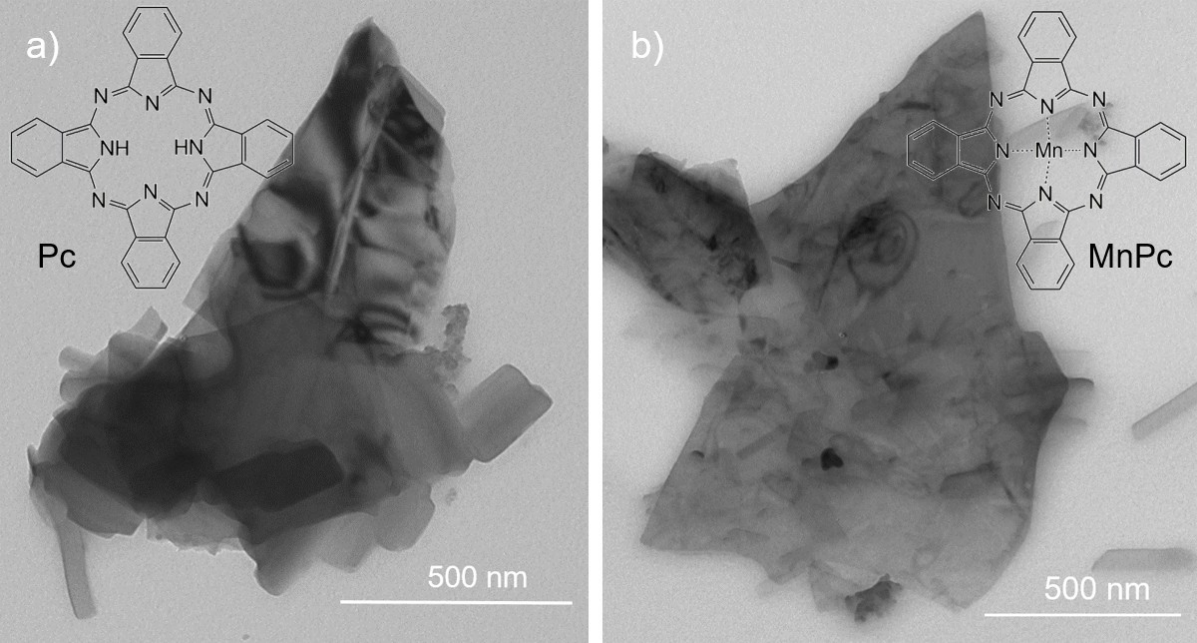
TEM images of the hybrid a) Pc‐bP and b) MnPc‐bP with inset of the structure of phthalocyanine (Pc) and manganese phthalocyanine (Mn‐Pc) respectively.

The Pc‐bP and MnPc‐bP nanohybrids were prepared by exfoliating bP crystals in the liquid phase, using ultrasonication, in the presence of the dye. Electron microscopy confirmed the presence of bP nanosheets in both nanohybrids, as shown by TEM images in Figure 1 and SEM image in Figure S1. Additionally, EDX (Energy Dispersive X‐ray spectroscopy) was performed on different spots and area to assess the chemical composition, (Figure S2). The results indicate an almost homogeneous distribution of N, P, and Mn across the nanosheet surface, shown in both localised spots and larger area. The lateral dimensions of the flakes range from 300 nm to 3 μm and their thickness goes from a few layers (~4 nm) to multilayers, as evaluated from TEM images (Figures S3 and S4).

**Figure 2:**
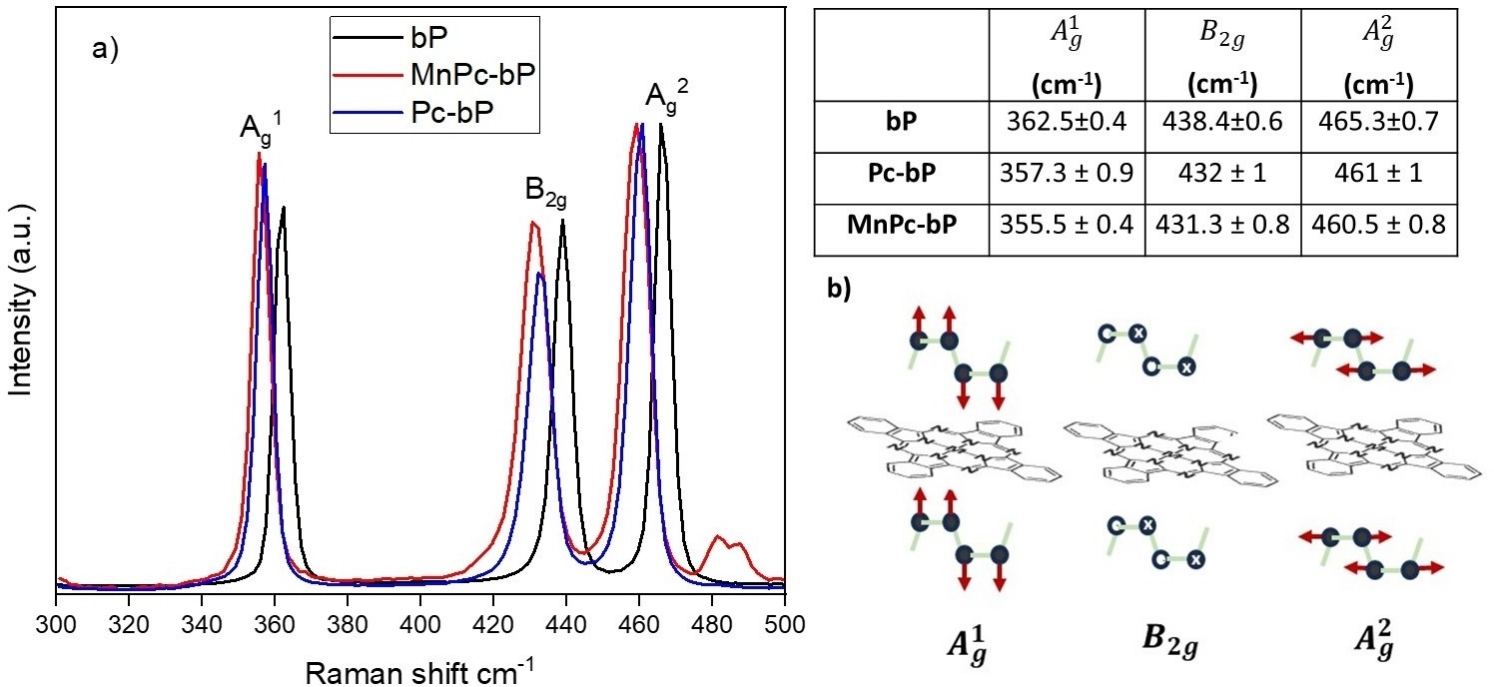
a) Raman spectra of bP, Pc‐bP and MnPc‐bP after being suspended in THF, deposited on a glass slide, and dried; inset Table: Raman shift values of the three characteristic vibrational modes; b) Schematic representation of possible allocation of phthalocyanines between the layers, impacting the vibrational modes.

**Figure 3:**
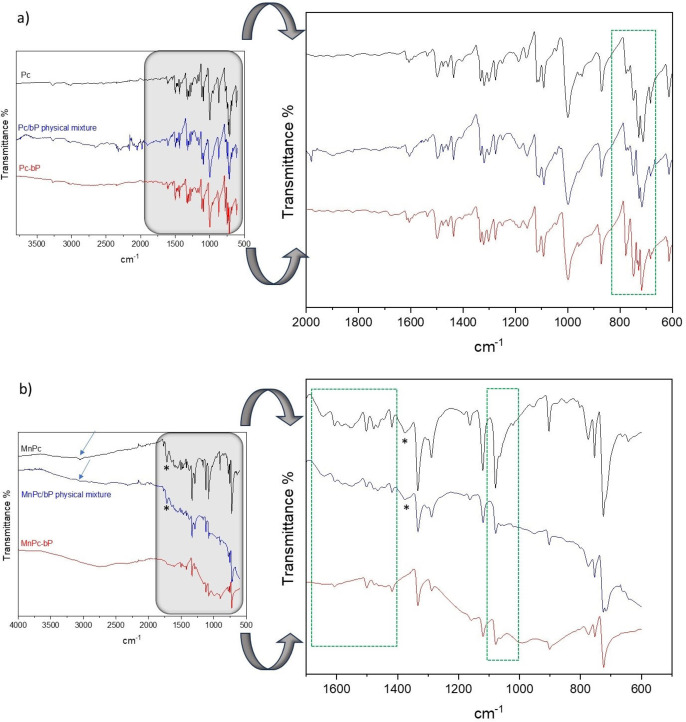
ATR‐FTIR spectra of a) Pc‐bP and b) MnPc‐bP nanohybrids with their corresponding physical mixtures Pc/bP and MnPc/bP and phthalocyanines. Spectra are translated for mere graphical needs. The symbol* is referred to an impurity.

The presence of both components (bP and phthalocyanine) in the nanohybrids was confirmed by Raman analysis (Figure 2) and ATR‐FTIR analysis (Figure 3).

The A_g_^1^, B_2g_, and A_g_^2^ vibrational modes of bare bP nanosheets^[10]^ are located at 362, 438 and 465 cm^−1^ as indicated by the black spectrum in Figure 2a. In the nanohybrids the peaks A_g_^1^, B_2g_ are shifted to lower wavenumbers by approximately 5–7 cm^−1^ (Figure 2a and inset Table). This red shift can be ascribed to a change in van der Waals interlayer forces, which are dominant along the stacking direction of bP. Going from the bulk to few‐layer bP results in P atoms oscillating within the layer with greater energy, since the covalent intralayer P bonds are stiffer than the interlayer van der Waals interactions and this corresponds to higher Raman shifts.[^10^, ^11^, ^12^] This contrasts with the experimental evidence we observed. Moreover, among the three vibrational modes of bP, A_g_^2^ is the most sensitive to the number of layers but, in our case, this mode appeared less affected by the functionalization, being red‐shifted of 4–5 cm^−1^. In agreement with the literature^[8]^ phthalocyanine molecules allocate between bP layers (Figure 2b) forming a heterostructure. This leads to different oscillations of P atoms falling perpendicularly to the layers, resulting in an increase in Raman scattering energy, especially in relation to the A_g_^1^ and B_2g_ modes, as was, indeed, observed. Such behavior was already proved featuring hybrids where the phthalocyanine molecules are adsorbed in a stacked planar geometry onto bP layers^[8]^ at intermolecular distances^[13]^ of ~3.2 and 3.3 Å with remarkable stability, as shown by calculated adsorption energies of up 2.75 eV for metal‐Pc.

The integrated intensity ratio of the A_g_^1^/A_g_^2^ is sensitively associated with sample oxidative degradation.^[14]^ We, therefore, analyzed the A_g_^1^/A_g_^2^ integrated intensity ratio of the Raman spectra for both nanohybrids, collecting 0.6±0.1 for MnPc‐bP and 0.7±0.1 for Pc‐bP. Since these values are ≥0.6 on average, even though they are close to the threshold,^[14]^ we can assume that significant oxidation events have not occurred with the functionalization. For comparison purposes, the Raman spectra of physical mixtures Pc/bP and MnPc/bP were collected and showed a clear overlap with the signals of bulk bP (see Figure S5), suggesting a very poor interaction between the components.

Figure 3 shows the ATR‐FTIR spectra of the two nanohybrids in comparison with their respective pristine phthalocyanines and physical mixtures between bP and the dye (samples Pc/bP and MnPc/bP). The IR spectra of phthalocyanines result from the envelope of vibrations of different structures, such as porphyrin macrocycles, isoindole, benzene, C−N=, and N−H. The main absorption bands are easily identifiable: in the range 600–2000 cm^−1^ there are bond bending vibrations with a distinctive fingerprint, whereas in the range 2000–4000 cm^−1^, there are bond stretching vibrations, generally less intense. In the case of Pc (Figure 3a), the stretching of the N−H bond can be found at 3272 cm^−1^ and the C−H stretching vibrations are observed between 3100 and 3000 cm^−1^. The absorptions of C=C at 1608 cm^−1^ and 1498 cm^−1^ are due to the benzene ring whose C−H bending can be found at 1435 and 1319 cm^−1^.

Porphyrin C=C stretching, and C−H bending are at 1458 and 1275 cm^−1^, respectively, while monosubstituted vinyl absorbs at 998 cm^−1^ (very intense vibrational mode diagnostic of metal‐free Pc).^[15]^ The bands C−N and C=N are in the range 1320–1310 cm^−1^. Out‐of‐plane deformation of porphyrin, C−H deformation, and C−H out‐of‐plane in porphyrin can be found at 613, 712, and 869 cm^−1^, respectively.^[16]^ These absorptions are also present in both the physical mixture Pc/bP and the nanohybrid Pc‐bP without substantial changes, with only weak variations in the region of C−H deformation below 800 cm^−1^‐ (see the green dotted box). This suggests that most of the Pc contained in the hybrid sample does not establish strong interactions with bP.

As opposed to Pc, MnPc shows more interesting modifications in the ATR‐FTIR spectrum once is coupled with bP due to the interaction between MnPc and bP. In detail, Figure 3b presents a comparison between MnPc‐bP, physical mixture MnPc/bP and MnPc. The latter shows absorption peaks diagnostic of metal phthalocyanines[^17^, ^18^]: the symmetric stretching vibration in phenyl rings at 3053 cm^−1^, the C−C stretching vibration in pyrrole ring at 1636 cm^−1^, the aryl and in‐plane bending of C−H between 1552 and 1409 cm^−1^, the C−C and C−N isoindole stretching at 1331 and 1289 cm^−1^ respectively, the in‐plane C−H bending and in‐plane deformation at 1120 and 1076 cm^−1^, the C−H out of plane deformations at 902 and 753 cm^−1^, the C−N deformation at 774 cm^−1^ and the porphyrin macrocycle ring deformation at 719 cm^−1^. The N−H stretching is not present in agreement with the metal (Mn) substitution. The spectrum of MnPc/bP is perfectly superimposed on that of MnPc, suggesting that no interactions between the dye and bP occur by simply mixing bP and MnPc. The absorptions at 1714 and 1366 cm^−1^, present in both MnPc and MnPc/bP (evidenced by stars in Figure 3b), are not ascribable to MnPc and are possibly due to residues of the synthetic procedure of the dye as proved by their disappearance in the spectrum of MnPc‐bP whose synthesis involves several solvent cleaning steps.^[19]^ Going to the spectrum of the nanohybrid, MnPc‐bP, the main signals of the dye are still present. However, some interesting differences can be observed, particularly in the stretching vibration of the pyrrole ring (1630–1640 cm^−1^) and in the frequency range associated with the aryl and in‐plane bending of C−H between 1550 and 1400 cm^−1^. In addition, the shape and intensities of the absorptions in the characteristic C−H bending zones are different with particular emphasis to the signal at 1076 cm^−1^, whose intensity slightly decreased (both regions of interest are evidenced in Figure 3b by a green box with a dotted line). These data suggest the occurrence of a localized interaction of bP with the aromatic and heteroaromatic rings, as confirmed by the reduction of C−H stretching intensity (see arrows in Figure 3b).

**Figure 4:**
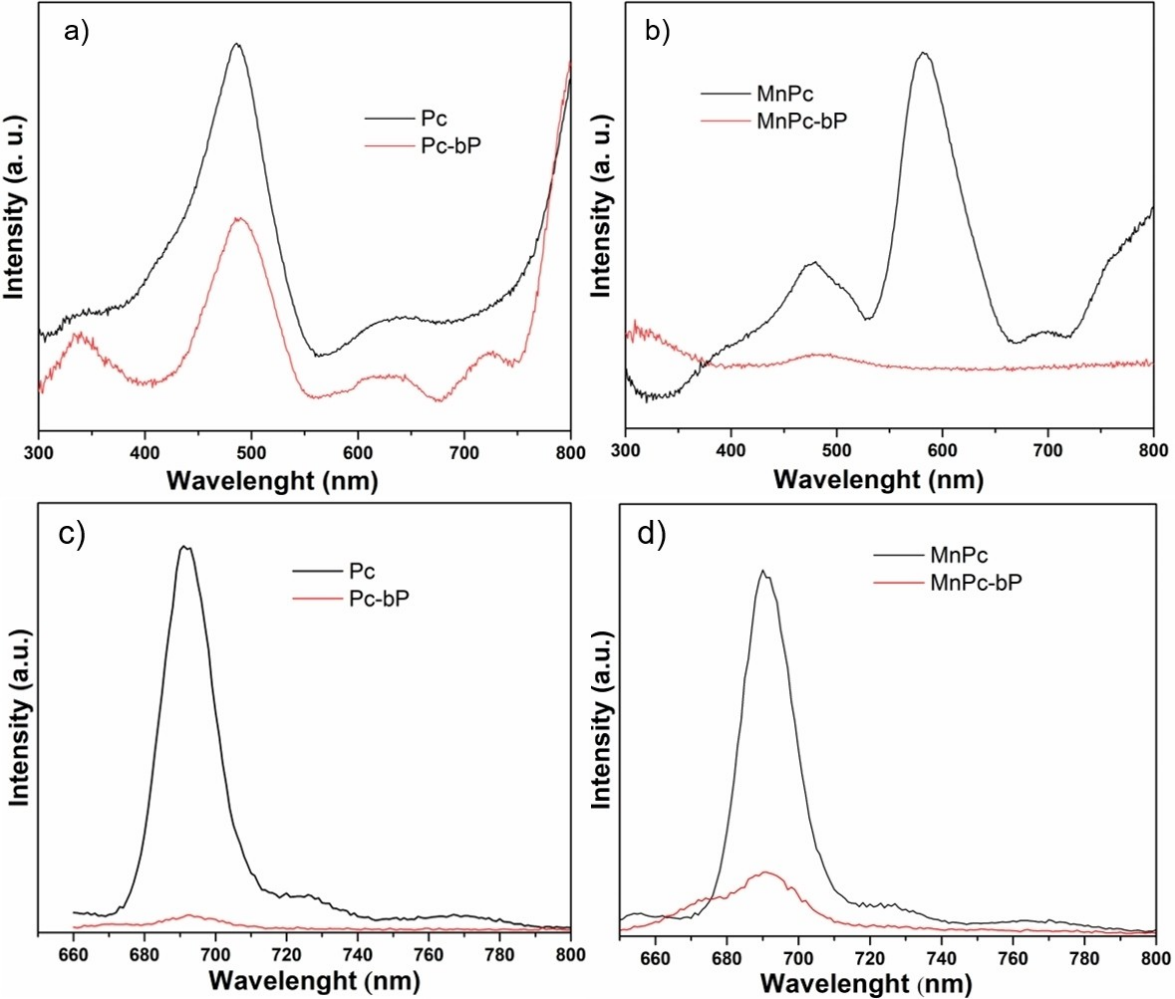
UV‐Vis DRS spectra of a) Pc and Pc‐bP and b) MnPc and MnPc‐bP and fluorescence spectra in DMF of c) Pc and Pc‐bP and d) MnPc and MnPc‐bP.

The UV‐Vis Diffuse Reflectance (UV‐Vis DRS) spectra were measured for both nanohybrids Pc‐bP and MnPc‐bP and compared with the respective pristine dye, as shown in Figure 4a) and b). As known from the literature,[^20^, ^21^] manganese phthalocyanine shows typical electronic spectra with three absorption regions: one is in the UV at about 300–400 nm (B band or Soret band), the second absorption is located between 470 and 560 nm and is induced by the charge‐transfer (CT) exciton of unsaturated manganese ions at the molecular center, the last one is the Q‐band located in the visible region at 600–700 nm. The latter is attributed to π–π* transition from the highest occupied molecular orbital (HOMO) to the lowest unoccupied molecular orbital (LUMO) of the Pc ring. The B band arises from the deeper π levels to the LUMO.[^22^, ^23^]

In Figure 4a), the UV‐Vis spectrum of the Pc‐bP nanohybrid shows a pattern similar to the parent dye, indicating that there is no relevant electronic interaction between the two components. In contrast, for MnPc‐bP, the formation of the nanohybrid is accompanied by an extensive broadening of the Q‐band and B band, as shown in Figure 4b). This suggests an electronic interaction in the ground state with bP playing as a quencher of the electronic excited states of the phthalocyanine moiety.[^24^, ^25^]

Excited state photoluminescence studies have been conducted to probe the photoinduced charge transfer process between bP and phthalocyanines. The steady‐state fluorescence spectra of the native dyes reveal a strong fluorescence intensity with a peak maximum at λ=691 nm and 689 nm for Pc and MnPc respectively, meanwhile going to the corresponding nanohybrids a clear reduction of emission intensity is observed, see Figure 4c) and d), which can be associated to an electron transfer between the phthalocyanine ring and bP.^[7]^

To disclose the oxidation state of phosphorus in the newly synthetized samples, Pc‐bP and MnPc‐bP, P 2p core level XPS spectra were measured and compared with that of pristine bP (see Figure S6). Meanwhile in the latter sample it is observed the characteristic doublet of elemental phosphorus^[26]^ with the P 2p_3/2_ and P 2p_1/2_ peaks located at 129.7 and 130.6 eV respectively, the samples functionalized with phthalocyanine show a high degree of oxidation, as testified by the presence of a broad peak centered at 134.5 eV, that accounts for oxidized phosphorus species, as P−O and P=O.^[26]^ With respect to the Raman spectra of Figure 2a, the limited probing depth of X‐ray photoemission suggests that the initiation of oxidation is restricted to the topmost layers of the nanohybrids. Interestingly, in the case of MnPc‐bP the amount of oxidized P species is lower than in Pc‐bP, seemingly the stronger interface interactions between MnPc and bP help to protect the bP surface from unavoidable ambient oxidation.^[27]^

Due to the very low content of manganese (0.56 wt%) in MnPc‐bP as assessed by ICP analysis, Mn could not be detected by X‐ray photoemission spectroscopy. Therefore, a more sensitive technique such as Near Edge X‐ray Absorption Fine Structure (NEXAFS) study was employed for further understanding of the molecule‐substrate interaction.

**Figure 5:**
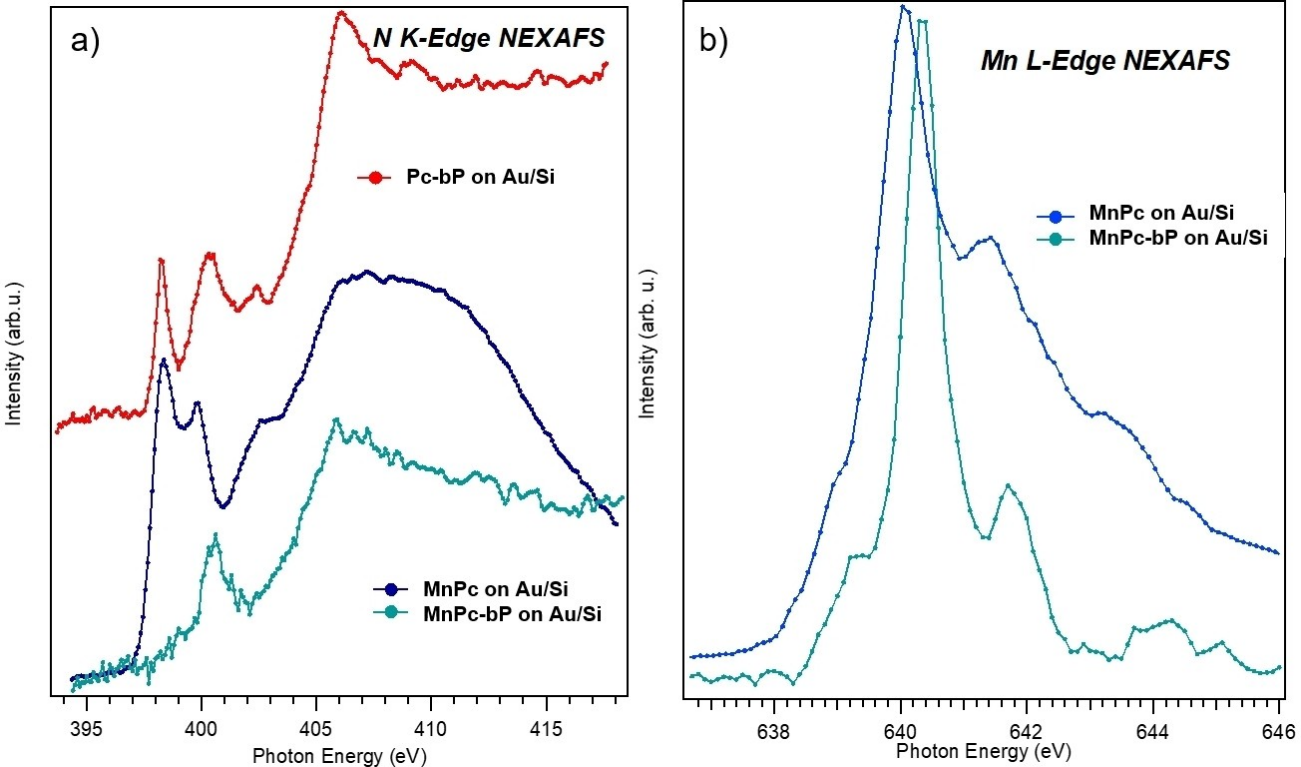
NEXAFS spectra measured at the a) N K‐edge and at the b) Mn L_3_‐edge on thin films on Au substrates of Pc‐bP, MnPc and MnPc‐bP.

The N K‐Edge spectra (Figure 5a) show multiple absorption peaks. They are commonly associated with transitions from the N 1s core level to the empty states located on the pyrrole macrocycles, mainly of π✶ symmetry, in the energy region 399–406 eV, and of σ✶ character, above 406 eV. In the case of the Pc‐bP nanohybrid, the measured transitions correspond to the ones reported in the literature^[28]^ for pristine Pc, suggesting Pc molecules are not strongly interacting with bP nanosheets, but most likely are physiosorbed on bP surface. A different situation was observed when the macrocycle contains Mn^2+^ since NEXAFS spectra of MnPc‐bP show a strong attenuation of the N‐K edge transition to the first empty state in comparison to pristine MnPc film, pointing that a charge redistribution has occurred. MnPc has two peaks at 398 and 399.5 eV and a broad band above 406 eV, (see Figure 5a). MnPc‐bP shows, beyond the band above 406 eV, only one peak at 401 eV in the region typical of π✶ transition, thus a shift of 1.5 eV to higher energy meaning N atoms much poorer from the electronic point of view.

Concerning the Mn L_3_ absorption edge, the MnPc film shows three distinct multiplet features (see Figure 5b) at 640, 641.2, and a shoulder at 643.2 eV, in agreement with the literature. For the nanohybrid MnPc‐bP, it is observed a variation of intensity, and there are mainly two peaks at 640.4 and 641.6 eV (both shifted of Δ=+0.4 eV), indicating electron doping of the molecules.^[29]^ The interaction of Mn^2+^ with bP affects the N atoms coordinated to the metal center, confirming that a charge redistribution process takes place from the dye MnPc towards P atoms of bP in agreement with UV‐Vis DRS and fluorescence data shown above. On the other hand, there is no sign of chemical interaction between Pc and bP; this means the metal cation's presence is decisive in establishing a strong electronic communication between Pc and bP nanosheet. The latter can be seen as a 2D polydentate phosphine ligand,^[30]^ where the lone pairs of P atoms coordinate via σ‐donation to the Mn^2+^ center and there is a π‐back donation from the metal to bP. This interaction justifies that Mn and its coordination environment, i.e. N atoms of the dye, are more electron‐poor compared to pristine MnPc.^[31]^ This can be rationalized considering MnPc molecules are arranged parallel to the surface of bP nanosheets to facilitate the interaction of bP lone pairs with the axial vacant sites in Mn^2+^, resulting in the formation of a vertical heterostructure.

## Conclusion

The paper describes the preparation and characterization of heterostructures between black phosphorus (bP) and phthalocyanine (Pc). The synthesis was performed via wet chemistry, by liquid phase exfoliation (ultrasonication) of bP crystals in the presence of the organic dye. The interaction between Pc and bP was studied using various techniques including Raman, FT‐IR, UV‐Vis, fluorescence, and NEXAFS. The results showed that bP exhibits electron‐donating behaviour towards the intercalated Pc. Changes in UV‐Vis DRS and IR spectra indicated a strong interaction between MnPc and bP, while the interaction pattern was unaltered in the case of Pc‐bP. Fluorescence spectra confirm strong π‐π interaction between the lone pairs of P atoms of bP and the aromatic ring of Pc leading to fluorescence quenching in the nanohybrids. NEXAFS measurements revealed that the interaction of pure Pc with bP can be seen as a physisorption governed by van der Waals forces while when the Pc molecule contains Mn^2+^, the interaction is mainly of a chemical nature due to coordinative bonds between P atoms lone pairs and empty d orbitals of Mn^2+^. All these findings provide an understanding of the intimate Pc‐bP and MnPc‐bP interfacial interaction that can be relevant for the development of 2D organic‐inorganic heterostructure with potential applications in various optoelectronics devices. In particular, the heterostructures should be optically sensitive and according to recent literature,^[32]^ in case of MnPc‐bP hybrid, owing to incorporation of magnetic cations it could be utilized for applications in spin‐related photodetection.

## Conflict of Interests

There are no conflicts of interest to declare.

1

## Supporting information

As a service to our authors and readers, this journal provides supporting information supplied by the authors. Such materials are peer reviewed and may be re‐organized for online delivery, but are not copy‐edited or typeset. Technical support issues arising from supporting information (other than missing files) should be addressed to the authors.

Supporting Information

## Data Availability

Experimental details and all the data that support the findings of this study are available in the supplementary material of this article.

## References

[1] J. Khan , R. T. M. Ahmad , J. Tan , R. Zhang , U. Khan , B. Liu , SmartMat 2023, 4, DOI: 10.1002/smm2.1156.

[2] A. Kumar , V. Kumar Vashistha , D. Kumar Das , Coord. Chem. Rev. 2021, 431, 213678.

[3] B.-D. Zheng , Q.-X. He , X. Li , J. Yoon , J.-D. Huang , Coord. Chem. Rev. 2021, 426, 213548. 10.1016/j.ccr.2020.213544 PMC7500364 32981945

[4] N. Sultana , A. Degg , S. Upadhyaya , T. Nilges , N. Sen Sarma , Mater. Adv. 2022, 3, 5557–5574.

[5] C. Wang , D. Niu , H. Xie , B. Liu , S. Wang , M. Zhu , Y. Gao , J. Chem. Phys. 2017, 147, DOI: 10.1063/1.4997724. 28810770

[6] P. K. Sarswat , M. L. Free , J. Electrochem. Soc. 2019, 166, B1–B8.

[7] Z. Liu , B. Zhang , N. Dong , J. Wang , Y. Chen , J. Mater. Chem. C Mater. 2020, 8, 10197–10203.

[8] F. Long , J. Zhou , L. Hu , S. Zhang , L. Qi , Y. Lu , H. Liang , L. Li , Y.-J. Zeng , J. Mater. Sci. 2021, 56, 13568–13578.

[9] S. Qiu , W. Yang , X. Wang , Y. Hu , Chem. Eng. J. 2023, 453, 139759.

[10] A. Impellizzeri , A. A. Vorfolomeeva , N. V. Surovtsev , A. V. Okotrub , C. P. Ewels , D. V. Rybkovskiy , PCCP 2021, 23, 16611–16622. 34319320 10.1039/d1cp02636d

[11] H. B. Ribeiro , M. A. Pimenta , C. J. S. de Matos , R. L. Moreira , A. S. Rodin , J. D. Zapata , E. A. T. de Souza , A. H. Castro Neto , ACS Nano 2015, 9, 4270–4276. 25752593 10.1021/acsnano.5b00698

[12] H.-Q. Bao , R.-B. Li , H.-D. Xing , C. Qu , Q. Li , W. Qiu , Appl. Sci. 2019, 9, 2198.

[13] D. Cortés-Arriagada , S. Miranda-Rojas , F. Cid-Mora , A. Toro-Labbé , J. Mol. Liq. 2021, 333, 115948.

[14] A. Favron , E. Gaufrès , F. Fossard , A.-L. Phaneuf-L'Heureux , N. Y.-W. Tang , P. L. Lévesque , A. Loiseau , R. Leonelli , S. Francoeur , R. Martel , Nat. Mater. 2015, 14, 826–832. 26006004 10.1038/nmat4299

[15] C. E. I. Aicha , Mahir , M. Hamouya , Mohamed , Int. J. Eng. Res. Technol. 2015, 4, 205–2010.

[16] D. Li , P. Zhang , S. Ge , G. Sun , Q. He , W. Fa , Y. Li , J. Ma , RSC Adv. 2021, 11, 31226–31234. 35496853 10.1039/d1ra04064b PMC9041327

[17] P. Sen , S. Zeki Yildiz , Res. Chem. Intermed. 2019, 45, 687–707.

[18] R. Seoudi , G. S. El-Bahy , Z. A. El Sayed , J. Mol. Struct. 2005, 753, 119–126.

[19] I. Yilmaz , New J. Chem. 2008, 32, 37–46.

[20] A. A. A. Darwish , S. Helali , S. I. Qashou , I. S. Yahia , E. F. M. El-Zaidia , Physica B Condens. Matter 2021, 622, 413355.

[21] L. Meng , K. Wang , Y. Han , Y. Yao , P. Gao , C. Huang , W. Zhang , F. Xu , Prog. Natural Sci.: Mater. Int. 2017, 27, 329–332.

[22] G. de la Torre , P. Vázquez , F. Agulló-López , T. Torres , Chem. Rev. 2004, 104, 3723–3750. 15352778 10.1021/cr030206t

[23] L. Breloy , O. Yavuz , I. Yilmaz , Y. Yagci , D.-L. Versace , Polym. Chem. 2021, 12, 4291–4316.

[24] M. de Miguel , M. Álvaro , H. García , Langmuir 2012, 28, 2849–2857. 22220928 10.1021/la204023w

[25] S. H. Noh , H. Park , W. Jang , K. H. Koh , M. Yi , J. M. Lee , S. Thirumalairajan , J. Y. Jaung , D. H. Wang , T. H. Han , Carbon N Y 2017, 119, 476–482.

[26] M. Caporali , M. Serrano-Ruiz , F. Telesio , S. Heun , A. Verdini , A. Cossaro , M. Dalmiglio , A. Goldoni , M. Peruzzini , Nanotechnology 2020, 31, 275708. 32235041 10.1088/1361-6528/ab851e

[27] B. Han , Z. Duan , J. Xu , Y. Zhu , Q. Xu , H. Wang , H. Tai , J. Weng , Y. Zhao , Adv. Funct. Mater. 2020, 30, DOI: 10.1002/adfm.202002232.

[28] M. V. Nardi , F. Detto , L. Aversa , R. Verucchi , G. Salviati , S. Iannotta , M. Casarin , PCCP 2013, 15, 12864. 23807700 10.1039/c3cp51224j

[29] G. Avvisati , P. Gargiani , C. Mariani , M. G. Betti , Nano Lett. 2021, 21, 666–672. 33356332 10.1021/acs.nanolett.0c04256

[30] L. Giusti , V. R. Landaeta , M. Vanni , J. A. Kelly , R. Wolf , M. Caporali , Coord. Chem. Rev. 2021, 441, 213927.

[31] X. Li , L. Xiao , H. Wang , J. Song , Q. Xu , M. Ye , J. Xu , Prog. Natural Sci.: Mater. Int. 2023, 33, 100–107.

[32] L. Hu , F. Liu , Q. Quan , C. Lu , S. Yu , L. Li , Adv. Funct. Mater. 2024, DOI: 10.1002/adfm.202409085.

