# Supplementary material for: Nanohybrids of 2D Black Phosphorus with Phthalocyanines: Role of Interfacial Interactions in Heterostructure Development
Source: Chemistry. 2024 Dec 10;31(5):e202403570. doi: 10.1002/chem.202403570 (PMC11753385; doi:10.1002/chem.202403570)
Supplement: Supplementary file 1 — Supporting Information [file CHEM-31-e202403570-s001.pdf]

Chemistry–A European Journal

Supporting Information

Nanohybrids of 2D Black Phosphorus with Phthalocyanines: Role of Interfacial Interactions in Heterostructure Development

Doriana Scittarelli, Serena Coiai, Francesca Cicogna, Stefano Legnaioli, Martina Dell'Angela, Alberto Verdini, Roberto Costantini, Manuel Serrano-Ruiz, Elisa Passaglia,* and Maria Caporali

SUPPORTING INFO

Experimental Details

Materials.

Unless otherwise stated, all the materials and the solvents were used as received without further purification. Red phosphorus, Sn, SnI_4, tetrahydrofuran (THF), anhydrous N,N-dimethylformamide (DMF), phthalocyanine (Pc), and manganese(II)-phthalocyanine (MnPc) were purchased from Merck.

Bulk black phosphorus was prepared through a minor modification[33] of the procedure reported in the literature[34]. Briefly, red phosphorus was introduced into a quartz tube under an inert atmosphere in the presence of tin and catalytic quantities of SnI_4. The tube was vacuum sealed and heated at a rate of 4.2 °C/min up to 406 °C, kept at this temperature for 2 h, and then heated up to 650 °C at a rate of 2.2 °C/min. The sample was left in the oven at 650 °C for 3 days and finally slowly cooled at 0.1 °C/min to room temperature to promote the formation of bP crystals with dimensions of approximately 2x3 mm.

Preparation of Pc- and MnPc-bP nanohybrids

The preparation procedure was optimized by repeated trials aimed at designing and experiencing the best conditions in terms of reproducibility. This includes three steps. First, into a 10 mL Schlenk flask, previously degassed, backfilled three times with nitrogen, and then left under nitrogen, about 7 mg of Pc or MnPc and anhydrous and degassed DMF (~1mL/1mg) were loaded and subjected to a sonication process carried out in an ice bath under inert atmosphere for 30 minutes to guarantee their complete dissolution. When the solution was homogeneous, bP crystals (in a 1:1 wt/wt ratio with respect to the dye) were added into the Schlenk flask and the sonication process was carried out for 5 hours to exfoliate bP in the presence of Pc (or MnPc). Finally, after a centrifugation step at 14000 rpm and solvent elimination, the solid product was washed two times with DMF, one time with THF and then dried in a vacuum oven. The procedure was repeated twice by recovering about 7.3-7.6 mg for MnPc-bP and 11.8-13.1 mg of Pc-bP hybrid. For comparison purposes two physical mixtures were prepared: Pc/bP and MnPc/bP (3/1 wt/wt) samples prepared by finely crushing bP crystals in an agate mortar with Pc and Mn Pc. The ensuing powders were analyzed using ATR-FTIR and Raman spectroscopies, and the outcomes were juxtaposed with the nanohybrids prepared through the method described above.

Instruments and Characterizations.

All ultrasonication processes used for the synthesis of the nanohybrids were carried out using a Hielscher Ultrasonic Processor UP200St (200W, 26kHz, commercial product Seneco Science) equipped with the Sonotrode S26d2 probe (tip diameter: 2mm) to the 55% amplitude adjustment and 2W power.

ICP-OES (Inductively Coupled Plasma-Optical Emission Spectroscopy) analyses were carried out with an Optima 8000 ICP-OES (Perkin Elmer) operating at 1500W and equipped with autosampler S10, MiraMist® Nebulizer (Perkin Elmer) and cyclonic chamber. Argon (420.069 nm) was used as the internal standard. Mn

was examined at 257.610 nm wavelength. The hybrid sample was analyzed after proper digestion in H_2O_2 and HNO_3. Metal content was determined by comparison with calibration curves obtained by commercial standard solutions (Fluka TraceCERT©) properly diluted in 2% HNO_3. The content of Mn was assessed as (0.56 wt%).
Transmission Electron Microscopy studies were carried out at Ce.ME CNR (Sesto Fiorentino, Italy) using a Philips CM12 electron microscope operating at an accelerating voltage of 80 kV. A few drops of each sample were suspended in tetrahydrofuran, placed on the TEM copper/carbon grid, dried under a stream of nitrogen, and measured.
Scanning Electron Microscopy experiments were carried out at Ce.M.E CNR (Sesto Fiorentino, Italy). The samples were analyzed using a Gaia 3 (Tescan s.r.o, Brno, Czech Republic) FIB-SEM (Focused Ion Beam-Scanning Electron Microscope) operating at the voltage of 5 kV and in high-vacuum mode and with secondary electron (SE) detector. Gaia 3 is equipped with an EDS-X-ray microanalysis system (EDX, AMETEK, USA) TEAM EDS Basic Software Suite TEAM™. A few drops of the sample suspended in tetrahydrofuran were placed on the TEM copper/carbon grid, dried under a stream of nitrogen, and measured.
The micro-Raman analysis was performed using a Renishaw Raman RM 2000 instrument equipped with an 1800 grooves/mm diffraction grating, a CCD detector, and a 50× lens NPLAN. The instrument has a Nd:YAG laser source at λ = 532 nm wavelength. The samples were analyzed as powder or after deposition of THF suspension on glasses and the spectra were recorded on different portions of each sample.
Attenuated Total Reflection Infrared (ATR-FT-IR) spectra were recorded with a Fourier Transform Spectrometer Perkin Elmer Spectrum Two equipped with a diamond crystal. Spectra were registered over the wavenumber range of 650-4000 cm^{-1} with a resolution of 4 cm^{-1} using 32 scans.
The UV-Vis diffuse reflectance spectra were measured on the solid samples with a Shimadzu UV-2600 spectrometer using an integrating sphere with $BaSO_4$ as a reference material.
Fluorescence measurements were carried out by using a Horiba FluoroMax-4 spectrofluorometer (emission spectra: λexc = 653, 620 and 616 nm, front entrance slit = 2 nm, front exit slit = 2 nm). The samples, suspended in DMF, were collected by using supernatants of centrifuged (14,000 rpm) fractions. The concentration of the Pc and MnPc was C = 1×10^{-6}M.
X-Ray Photoemission Spectroscopy (XPS) and Near Edge X-Ray Absorption Fine Structure (NEXAFS) measurements were carried out at the ANCHOR-SUNDYN endstation [35] of the ALOISA beamline of Elettra Synchrotron. Thin films of Pc, Pc-bP, MnPc, MnPc-bP and bP (as a reference) were prepared on Au substrate via drop-casting of the sample suspended in acetone. All spectra have been acquired by using a Phoibos 150 electron analyzer (Specs). NEXAFS spectra have been measured in Auger Yield mode. The X-ray photons were linearly polarized, with the electric field vector at the magic angle to the surface plane to average on molecular geometries.

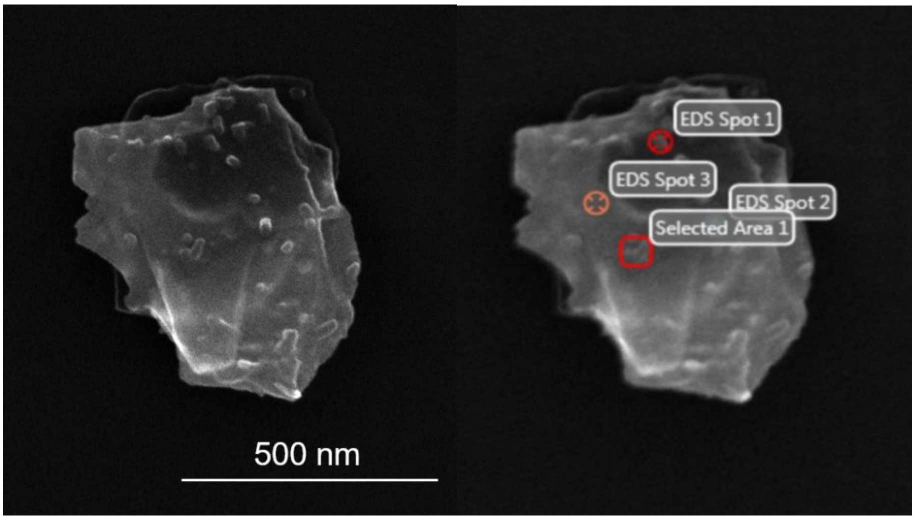

Figure S1. SEM image of MnPc-bP (on the right spotted area selected for EDAX analysis).

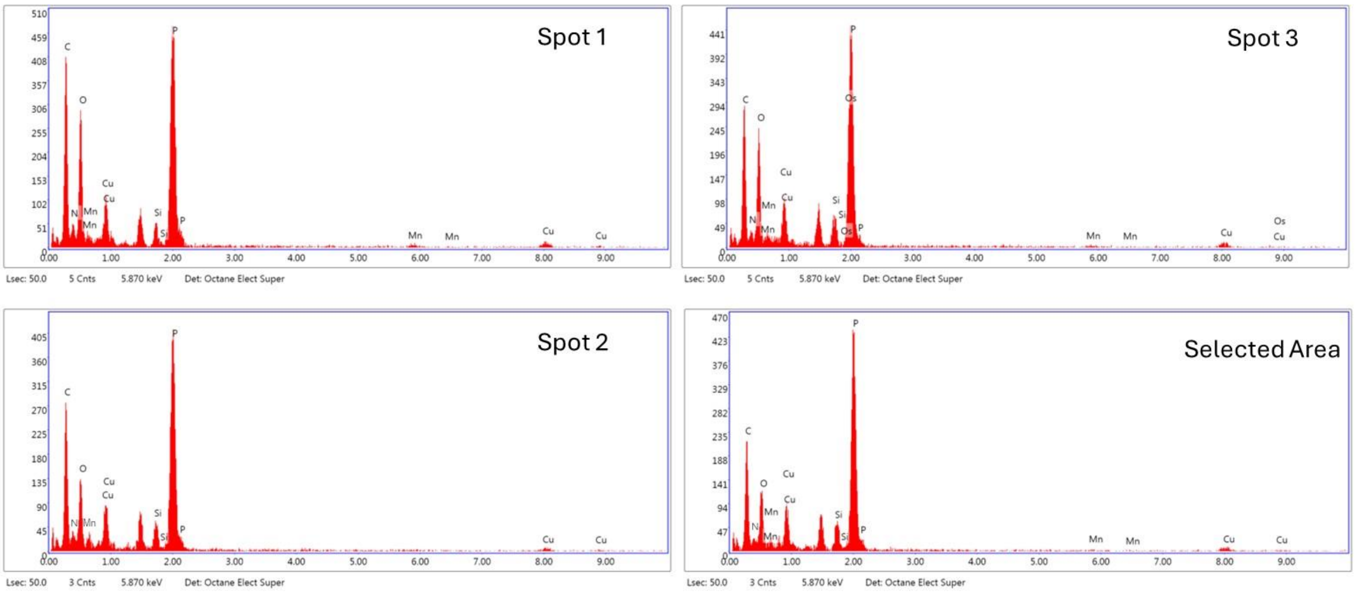

Figure S2. Energy dispersive X-ray spectrum (EDAX) of MnPc-bP measured on the selected spots and area of the flakes shown above in Figure S1. The peak of Cu is due to carbon/copper grid used for the deposition of the sample. The peak of Si is due to an impurity (silicon grease).

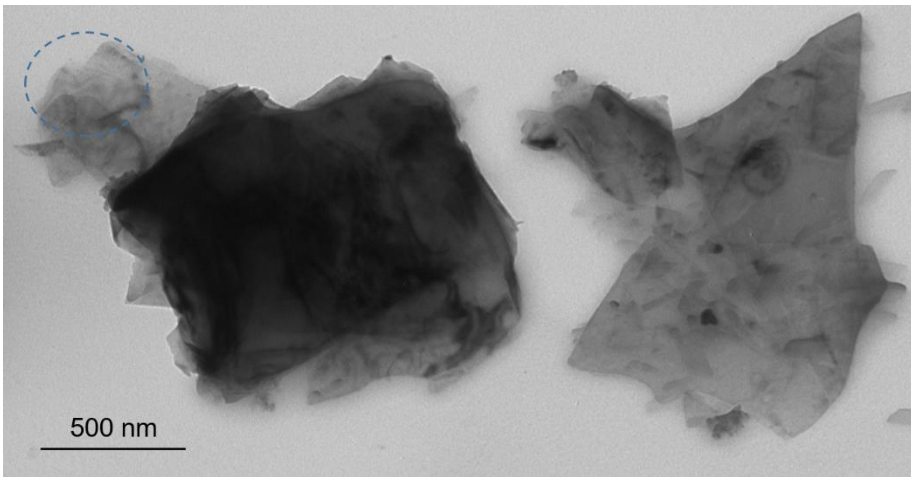

Figure S3. Bright field TEM image of MnPc-bP. Scale bar: 500 nm. Looking at the circled edges, it is possible to count 6 layers, considering the interlayer distance in black phosphorus is 0.53 nm, plus the thickness of each layer, in total we have a thickness of ~4.2 nm.

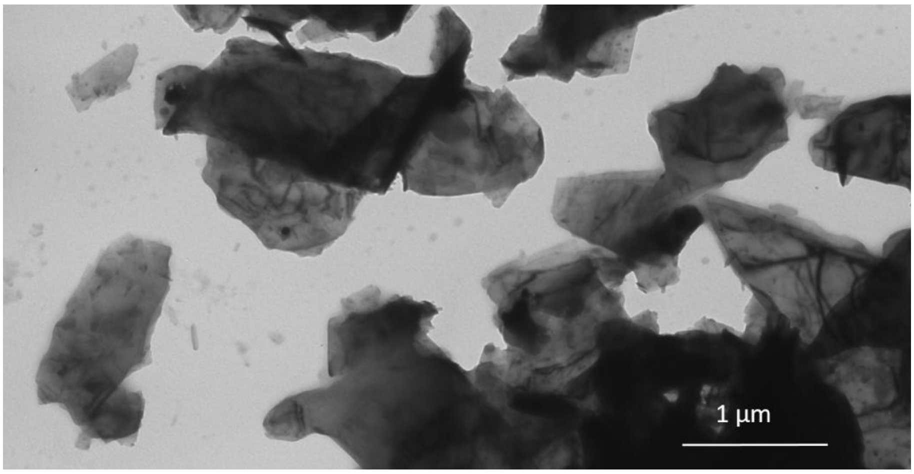

Figure S4. Panoramic view of the sample MnPc-bP by TEM imaging. Scale bar: 1µm.

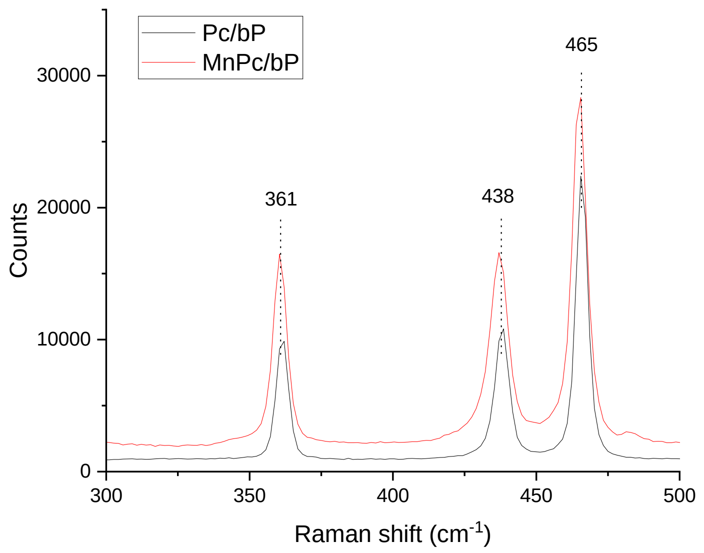

Figure S5. Raman spectra of physical mixtures between Pc and MnPc with bP; samples Pc/bP and MnPc/bP.

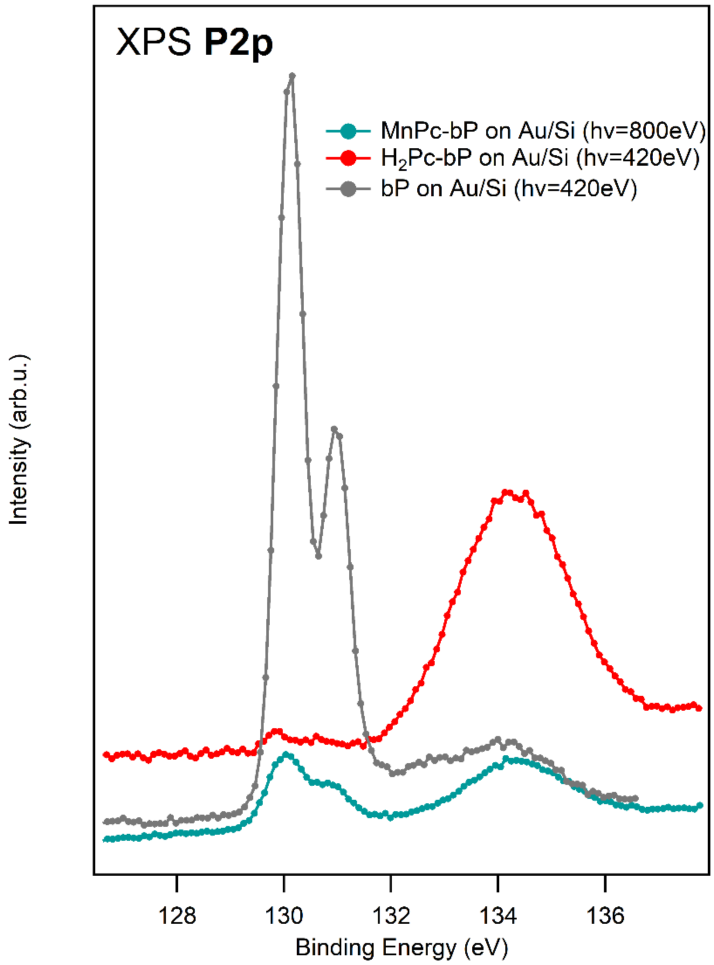

Figure S6. P 2p core-level XPS spectrum of pristine bP, Pc-bP and MnPc-bP.

[33] N. Hemsworth, V. Tayari, F. Telesio, S. Xiang, S. Roddaro, M. Caporali, A. Ienco, M. Serrano-Ruiz, M. Peruzzini, G. Gervais, T. Szkopek, S. Heun, *Phys Rev B* **2016**, *94*, 245404.

[34] M. Köpf, N. Eckstein, D. Pfister, C. Grotz, I. Krüger, M. Greiwe, T. Hansen, H. Kohlmann, T. Nilges, *J Cryst Growth* **2014**, *405*, 6–10.

[35] R. Costantini, M. Stredansky, D. Cvetko, G. Kladnik, A. Verdini, P. Sigalotti, F. Cilento, F. Salvador, A. De Luisa, D. Benedetti, L. Floreano, A. Morgante, A. Cossaro, M. Dell'Angela, *J Electron Spectros Relat Phenomena* **2018**, *229*, 7–12.
